# The Frmpd3 Protein Regulates Susceptibility to Epilepsy by Combining with GRIP and GluA2

**DOI:** 10.3390/cimb47040225

**Published:** 2025-03-26

**Authors:** Yan Jia, Jinqiong Zhan, Pengcheng Huang, Xiaobing Li, Daojun Hong, Xi Lu

**Affiliations:** 1 Department of Neurology, The First Affiliated Hospital, Jiangxi Medical College, Nanchang University, Nanchang 330047, China; 2 Institute of Neurology, Jiangxi Academy of Clinical Medical Science, The First Affiliated Hospital, Jiangxi Medical College, Nanchang University, Nanchang 330047, China; 3 Rare Disease Center, The First Affiliated Hospital, Jiangxi Medical College, Nanchang University, Nanchang 330047, China; 4 Key Laboratory of Rare Neurological Diseases of Jiangxi Provincial Health Commission, Jiangxi Medical College, Nanchang University, Nanchang 330047, China; 5 Biological Psychiatry Laboratory, Jiangxi Mental Hospital & Affiliated Mental Hospital of Nanchang University, Nanchang 330047, China; 6 Jiangxi Provincial Clinical Research Center on Mental Disorders, Jiangxi Mental Hospital, Nanchang 330047, China; 7 Nanchang City Key Laboratory of Biological Psychiatry, Jiangxi Mental Hospital, Nanchang 330047, China

**Keywords:** epilepsy, scaffold protein, AMPAR, GluA2, GRIP

## Abstract

Frmpd3 (FERM and PDZ Domain Containing 3), a scaffold protein potentially involved in excitatory synaptic function, has not been thoroughly characterized in terms of its expression and functional role in vivo. Here, we investigated the distribution of Frmpd3 in the central nervous system and its potential regulatory role in epilepsy, a neurological disorder characterized by disrupted excitatory–inhibitory balance. The distribution of Frmpd3 throughout the mouse brain was investigated by immunofluorescence. Western blotting was conducted to examine potential alterations in Frmpd3 protein expression in the hippocampus of a pentylenetetrazol (PTZ)-induced chronic epilepsy model. Using stereotaxic techniques, we delivered Frmpd3 siRNA-AAV9 into the hippocampal CA1 region to achieve targeted protein knockdown. Then, the functional consequences of Frmpd3 depletion were assessed through behavioral observations and electrophysiological recordings in PTZ-treated mice. Finally, protein–protein interactions were investigated using immunoprecipitation and Western blot analysis. Immunofluorescence analysis revealed Frmpd3 expression in cortical, hypothalamic, cerebellar, and hippocampal neurons of adult mice. Subcellular localization studies demonstrated predominant distribution of Frmpd3 in the excitatory postsynaptic density (PSD) of hippocampal CA1 neurons, with additional expression in inhibitory neurons. Quantitative analysis showed significantly elevated Frmpd3 protein levels in the hippocampus of PTZ-induced epileptic mice compared to controls. Frmpd3 knockdown in the CA1 region resulted in the following: (1) reduced seizure frequency, (2) prolonged seizure latency, and (3) decreased incidence of PTZ-induced generalized seizures. Local field potential (LFP) recordings demonstrated that seizure amplitude tended to be reduced, and epileptic discharge durations tended to be shorter in Frmpd3-depleted mice compared to controls. Furthermore, we observed decreased membrane expression of the AMPA receptor GluA2 subunit in the hippocampus of Frmpd3 knockdown mice. Molecular interaction studies revealed that Frmpd3 forms complexes with glutamate receptor-interacting protein (GRIP) and GluA2. Our findings identify Frmpd3 as a novel regulatory scaffold protein that modulates epileptic susceptibility through molecular interactions with GRIP and GluA2. The underlying mechanism appears to involve Frmpd3-mediated regulation of GluA2 trafficking from the cytoplasm to the membrane, ultimately enhancing neuronal excitability through increased membrane expression of GluA2-containing AMPA receptors.

## 1. Introduction

Epilepsy is a common neurological disease characterized by long-term abnormal neuron discharge leading to corresponding neurological damage [1]. Although scientists have developed approximately 20 kinds of antiseizure medications (ASMs) for clinical use, approximately 20–40% of patients with epilepsy are not sensitive to ASMs, resulting in the development of drug-resistant epilepsy (DRE) [2]. However, the exact etiology of epilepsy and its pathogenesis are not yet fully understood.

α-amino-3-hydroxy-5-methyl-4-isoxazole-propionic acid receptor (AMPAR) and N-methyl-D-aspartate receptor (NMDAR) are two ionotropic glutamate receptors commonly related to excitatory synaptic activity. AMPAR, a ligand-gated channel receptor, mediates the majority of fast excitatory synaptic transmission in the pathogenesis of epilepsy [3,4]. Given the critical role of AMPAR in epilepsy, ASMs targeting AMPAR, such as perampanide, have been used in clinical treatment [5,6]. AMPAR is a heterotetramer composed of four subunits, namely, GluA1-A4. The GluA2 subunit exhibits characteristically low calcium permeability, a critical property that is essential for maintaining the proper biological function of AMPAR [7]. The trafficking of AMPAR between the cytoplasm and the postsynaptic membrane is a crucial process that regulates its distribution on the synaptic surface [8]. AMPAR-interacting proteins play a pivotal role in AMPAR transport mechanism.

The scaffolding proteins containing PDZ domains are well-recognized as AMPAR-interacting proteins. The PDZ domain, highly conserved from yeast to humans, comprises 90 amino acids that form two α-helices and six β-folds [9]. The structure of the PDZ domain identifies the C-terminal amino acid sequence of ligand proteins, which are widely distributed in the postsynaptic membrane of excitatory neurotransmitter receptors. It plays roles spanning cytoplasmic transport to anchoring cell membrane receptors [10]. Recombinant glutamate receptor-interacting protein (GRIP) is a well-characterized PDZ protein involved in AMPAR transport [11]. GRIP is a scaffolding protein that contains seven PDZ domains and a small globular protein–protein interaction domain. The PDZ domains of GRIP directly bind to the C-termini of the AMPAR GluA2 and GluA3 subunits, regulating the transport of AMPAR between the cytoplasm and the cell membrane, which plays an important role in excitatory synaptic transmission and synaptic plasticity [11,12].

Frmpd3 is a novel scaffold protein containing a PDZ domain and a FERM domain. While its precise distribution and biological functions in the central nervous system remain unclear, its multi-domain structure suggests a potential critical role in neuronal excitation. Hu et al. demonstrated via co-immunoprecipitation that Frmpd3 interacts with the scaffolding protein Homer1 in excitatory synapses in vitro [13]. Spiegel et al. observed that Npas4 selectively upregulates Frmpd3 expression in inhibitory neurons [14]. Notably, the homologous scaffold protein Frmpd2 was shown to interact with the NMDAR subunit NR2A through its PDZ domain [15]. These findings support the hypothesis that Frmpd3 may utilize its PDZ domain to engage with neurotransmitter receptors, potentially regulating receptor trafficking and contributing to neuronal hyperexcitability and epileptogenesis.

However, the distribution and function of Frmpd3 in the central nervous system remain unclear, particularly its role in epilepsy. In this study, we reveal Frmpd3 protein expression in specific mouse brain regions and its probable functions in epilepsy. We demonstrate that Frmpd3 modulates epileptic processes through interactions with the AMPAR GluA2 subunit and the AMPAR-interacting protein GRIP.

## 2. Materials and Methods

### 2.1. Pentylenetetrazol-Kindled Chronic Epilepsy Mouse Models

Adult wild-type C57B/L6 mice were intraperitoneally injected with pentylenetetrazole (PTZ, 35 mg/kg) every other day for one month. After injection, the samples were observed with a video camera for at least one hour [16]. Behavioral seizure scores were evaluated according to the Racine standard scale: grade 0, arrest and normal behavior; grade 1, facial twitches (nose, lips, and eyes); grade 2, chewing and head nodding; grade 3, forelimb clonus; grade 4, rearing and falling on the forelimbs and hind limb clonus; and grade 5, rearing and falling on the side or back. Only grades 4 and 5 seizures were considered fully kindled seizures and were included in the number of seizures. The days until full kindling were recorded as the latency period.

### 2.2. Immunofluorescence Staining

Fresh animal brain tissues were postfixed for 24 h at 4 °C. Floating slices (35 μm thick) were permeabilized with 2% Triton X-100 for 30 min at room temperature and then incubated and blocked in an immunofluorescence blocking solution. The slices were incubated with primary antibodies against Frmpd3 (rabbit, Thermo Fisher, Waltham, MA, USA), and NeuN (mouse, Abcam, Waltham, MA, USA), Frmpd3 (rabbit, Thermo Fisher, Waltham, MA, USA), and GFAP (mouse, Cell Signaling Technology, Waltham, MA, USA), Frmpd3 (rabbit, Thermo Fisher, Waltham, MA, USA) and PSD95 (mouse, Cell Signaling Technology, Waltham, MA, USA), and Frmpd3 (rabbit, Thermo Fisher, Waltham, MA, USA), and GAD67 (mouse, Abcam, Waltham, MA, USA) overnight at 4 °C, followed by incubation with secondary fluorescent-conjugated antibodies for 4 h at room temperature. 4′,6-Diamidino-2-phenylindole (DAPI) was used for nuclear staining. Images were captured under a confocal microscope (Leica; Wetzlar, Germany).

### 2.3. Western Blot

The hippocampus of PTZ-kindled mice and control mice were collected and extracted for total protein analysis via a RIPA protein extraction kit (Beyotime Biotechnology, Shanghai, China). The protein mixture was then boiled at 95 °C for 5 min, after which the protein concentrations were calculated via an enhanced bicinchoninic acid (BCA) protein assay kit (Beyotime Institute of Biotechnology, Shanghai, China). The proteins were separated on 8% SDS-PAGE gels, and the separated target proteins were immunoblotted with primary antibody Frmpd3 (rabbit, Thermo Fisher, Waltham, MA, USA), β-actin (mouse, Proteintech, Wuhan, China), and α-Tubulin (mouse, Proteintech, Wuhan, China), anti-GluA2 (rabbit, Proteintech, Wuhan, China), anti-GRIP1 (rabbit, Proteintech, Wuhan, China), anti-GluN2B (rabbit, Proteintech, Wuhan, China), anti-ATP1A1 (mouse, Proteintech, Wuhan, China) overnight at 4 °C. The blots were then incubated with HRP-conjugated secondary antibodies. The blots were scanned and quantified via a fusion imaging system.

### 2.4. Intrahippocampal Injection of AAV

Healthy adult C57/BL6 male mice weighing 25 ± 2 g were obtained from Henan Skobes Biological Co., Ltd., Zhengzhou, Henan, China. Intrahippocampal injections of adeno-associated virus (AAV) were administered via a stereotaxic apparatus (RWD Co., Ltd., Shenzhen, China). AAV carrying shRNA targeting mouse Frmpd3 (NM_001320946.1) was from OBiO Technology (Shanghai) Corp., Ltd., Shanghai, China. Mouse shRNA sequence was 5′-GCAGCCTCATTGAGACCTT-3′. Briefly, the mice were anesthetized with pentobarbital (80 μg/g, intraperitoneally) and then placed in a stereotaxic apparatus. AAV9 vectors (siCtrl, siFrmpd3) were injected into the CA1 hippocampal region of the mice (Paxinos Mouse Brain Atlas; coordinates from bregma; anteroposterior (AP): −1.7 mm; mediolateral (ML): ±1.3 mm; dorsoventral (DV): −1.7 mm) via a microliter syringe controlled by an injection pump. All AAV deliveries were performed bilaterally with a volume of 1 μL each at a flow rate of 0.1 μL/min. The needle was kept in place for 15 min after each injection. The injected mice were allowed to fully recover for 2 weeks to prevent infection, and recovery was followed by PTZ injection for behavioral observation.

### 2.5. LFP Recording

Surgery was performed 1 week prior to the recordings. Briefly, to record the hippocampal LFP (Local field potential, LFP), the mice were anesthetized and placed in a stereotaxic apparatus, and two stainless steel screws implanted in the anterior cranium served as the ground electrode. For LFP recording, the recording electrode was implanted into the prefrontal lobe (anteroposterior (AP): +1.7 mm; mediolateral (ML): −0.4 mm), and the reference electrode was implanted into the cerebellum. A typical seizure-like event is characterized by the disappearance of the electrophysiological rhythm connected to the A-M system 1800 acquisition system and the occurrence of a cluster of spontaneous paroxysmal discharges with amplitudes greater than 2 times the baseline and durations greater than 5 s.

### 2.6. Immunoprecipitation

Hippocampal tissue samples were lysed with RIPA lysis buffer (Beyotime Biotechnology, Shanghai, China) and supplemented with a protease inhibitor cocktail (Thermo Fisher, Waltham, MA, USA) at 4 °C for 30 min. Lysates containing equal amounts of protein were incubated with protein A/G magnetic beads (Beyotime Biotechnology, Shanghai, China) bound to anti-Frmpd3, anti-GluA2 (rabbit, Proteintech, Wuhan, China), anti-GRIP1 (rabbit, Proteintech, Wuhan, China), or anti-GluN2B (rabbit, Proteintech, Wuhan, China) antibodies at 4 °C overnight, according to the manufacturer’s instructions. After washing, the proteins were harvested by magnetic separation, and the magnetic beads were eluted by adding 100 μL of 1× SDS–PAGE loading buffer. Proteins were subsequently analyzed via Western Blotting.

### 2.7. Statistical Analysis

GraphPad Prism 9.0 software (San Diego, CA, USA) was used for statistical analysis. Experimental data are presented as either medians with interquartile ranges or means ± standard deviations (SDs). *p* < 0.05 was considered statistically significant. The normality of the data was analyzed via the Shapiro–Wilk test. When the variance of the dataset was significantly different, we used nonparametric statistical analysis. Two-tailed Student’s *t*-tests were used to compare two independent groups.

The workflow for fluorescence colocalization analysis includes the following: Defining regions of interest in Leica Las X to encompass colocalization signals; extracting intensity values from both channels along spatial/temporal axes and plotting synchronized curves; assessing curve synchrony via visual inspection; interpreting results where co-expression indicates synchronous curve trends, while no correlation reflects random fluctuations.

## 3. Results

### 3.1. Distribution of Frmpd3 Proteins in the Mouse Brain

**Figure 1:**
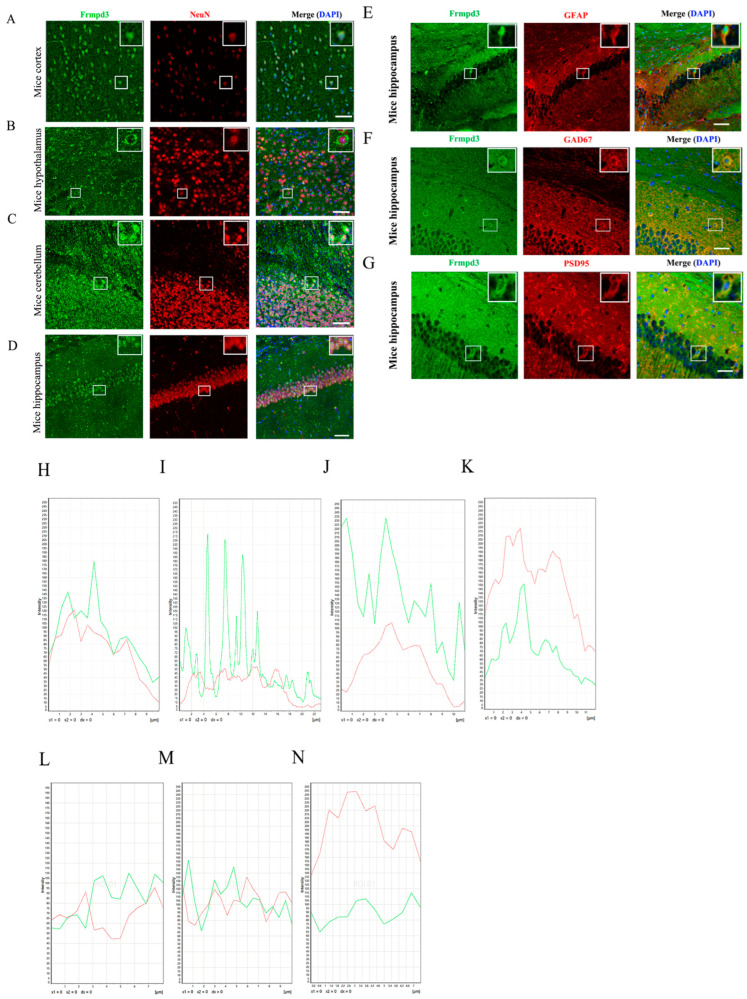
Distribution of the Frmpd3 protein in the adult mouse brain. In the adult mouse brain, the Frmpd3 protein was expressed in the cortex (**A**), hypothalamus (**B**), cerebellum (**C**), and hippocampal CA1 region (**D**) and colocalized with the neuronal marker NeuN. The Frmpd3 protein did not colocalize with the astrocyte marker GFAP (**E**) but colocalized with the inhibitory neuron markers GAD67 (**F**) and PSD95 in the dense region of the excitatory postsynaptic membrane (**G**). Green fluorescence indicates Frmpd3, red fluorescence indicates NeuN (neurons), GFAP (astrocytes), GAD67 (inhibitory neurons), PSD95 (excitatory postsynaptic density), and blue fluorescence indicates DAPI (nucleus), bar = 20 μm; (**H**–**N**) The co-expression quantification analysis of (**A**–**G**), *n* = 3.

As a scaffold protein, Frmpd3 has been speculated to be related to excitatory synaptic function, but the specific biological function of Frmpd3 in the nervous system is still unknown. We first observed the distribution of Frmpd3 in the mouse brain. The results of the immunofluorescence staining of mouse brain tissues revealed that Frmpd3 was expressed in the cortex, hypothalamus, cerebellum, and hippocampus (Figure 1A–D), among which the hippocampus is an important brain area related to the occurrence and development of epilepsy. Immunofluorescence staining of the hippocampal CA1 area revealed that Frmpd3 merged with NeuN (neuronal marker), as shown in Figure 1D, and did not merge with GFAP (astrocyte marker), as shown in Figure 1E. Frmpd3 merged with GAD67 (inhibitory neuron marker) or PSD95 (excitatory postsynaptic membrane marker) (Figure 1F,G). These findings indicated that Frmpd3 was located mainly in the neurons and might play a role in synaptic transmission.

### 3.2. The Expression of Frmpd3 Protein Increased in the Hippocampus of PTZ-Induced Epileptic Mouse Model

**Figure 2:**
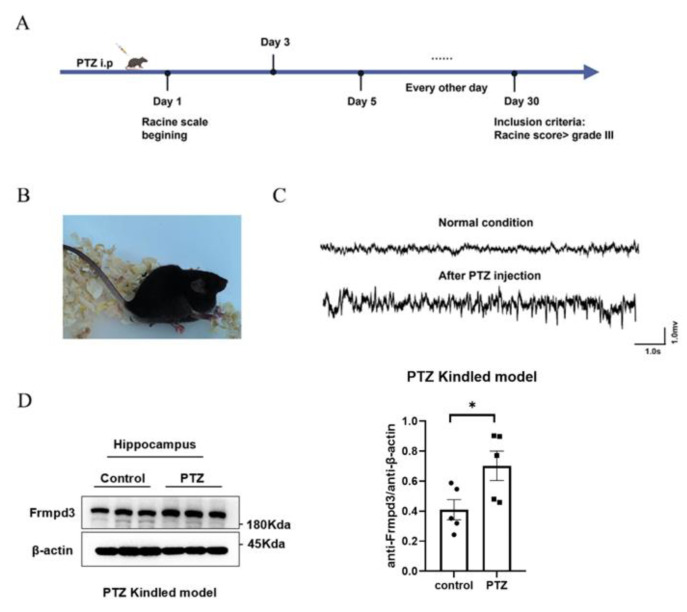
Increased Frmpd3protein expression was detected in the brain tissue of the PTZ-induced chronic epilepsy mouse model. (**A**) PTZ-induced flow chart of chronic epilepsy animal models. (**B**) Successful induction of seizures in adult mice, Racine score grade V, i.e., falls and tonic–clonic seizures. (**C**) Typical PTZ-induced chronic epilepsy seizures in mice to V attack field potential diagram (the horizontal axis represents time bar = 1.0 s, the vertical axis represents the amplitude bar = 1.5 mv). (**D**) Increased Frmpd3protein expression was found in the hippocampal tissue of the PTZ-induced chronic epilepsy mouse model (*n* = 5, unpaired two-tailed Student’s *t*-test, * *p* < 0.05). Frmpd3 (FERM and PDZ Domain Containing 3), PTZ (Pentylenetetrazole).

The immunofluorescence staining results revealed Frmpd3 protein expression in the PSD, suggesting that Frmpd3 in hippocampal neurons may be associated with epilepsy. To further explore the changes in Frmpd3 protein levels in epileptic brain tissue, we established a chronic epilepsy animal model via the intraperitoneal injection of PTZ in mice (Figure 2A). According to the Racine score, only mice with more than three consecutive grade IV or V seizures were included in the subsequent experiments (Figure 2B). These mice were used as successful models in the experimental group. The LFP results (Figure 2C) revealed typical epileptic discharge. We collected mouse hippocampal tissue for Western blot analysis, and the results revealed that compared with that in the control group, Frmpd3 protein expression in the experimental group was significantly greater (Figure 2D).

### 3.3. Hippocampal Downregulation of Frmpd3 Protein Expression Suppressed Seizure Activity in a PTZ-Induced Epileptic Mouse Model

**Figure 3:**
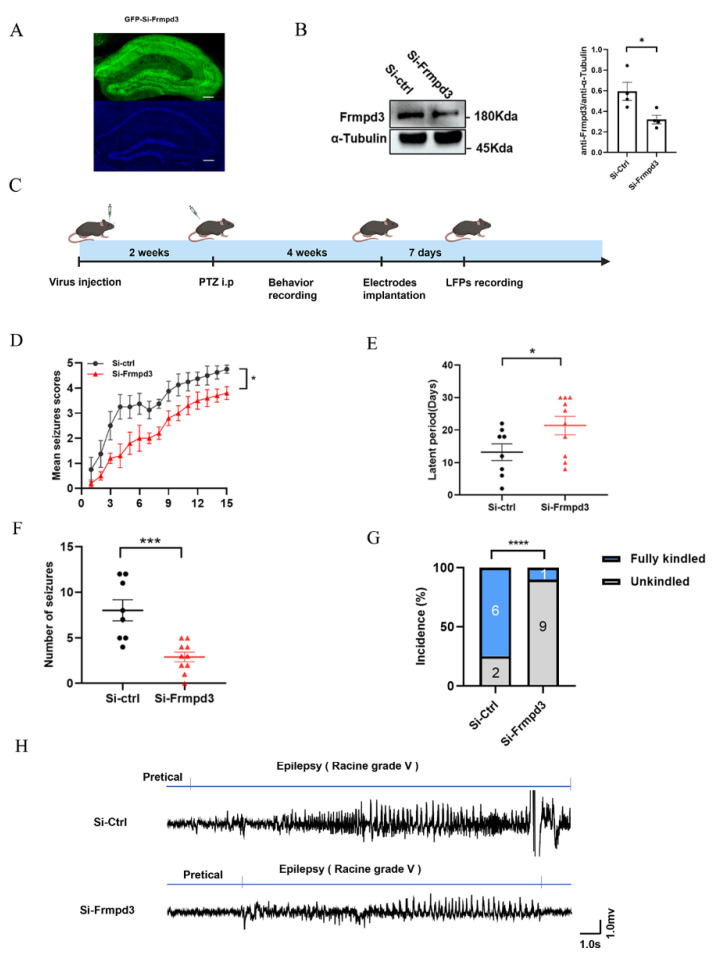
Downregulation of the Frmpd3 protein reduces epilepsy susceptibility. (**A**) Two weeks after AAV expression, immunofluorescence in the mouse hippocampal CA1 area was successful in which GFP-labeled AAV was transfected, bar = 150 microns. (**B**) Two weeks after AAV expression, the expression of Frmpd3 protein in the hippocampus of the Si-Frmpd3 group was significantly lower than that in the Si-ctrl group (*n* = 4; unpaired two-tailed Student’s *t*-test, * *p* < 0.05). (**C**) As shown in the flow diagram, a chronic epilepsy mouse model induced by intraperitoneal injection of PTZ was generated after AAV injection. Behavioral changes and field potential were analyzed. (**D**–**G**) The daily scores of seizures (**D**), ignition latency (**E**), and numbers of level IV and V seizures (**F**) were recorded after Frmpd3 knockdown in an animal model of chronic epilepsy induced by PTZ kindling (*n* = 8–10; unpaired two-tailed Student’s *t*-test; * *p* < 0.05, *** *p* < 0.001). The success rates of complete kindling (**G**) in the Si-Frmpd3 group and the Si-ctrl group were also quantified (*n* = 8–10, chi-square test, **** *p* < 0.0001). (**H**) Schematic representation of typical field potentials during Racine grade V seizures in the control and Si-Frmpd3 groups (the horizontal axis represents time bar = 1.0 s, and the vertical axis represents amplitude bar = 1.0 mv). Frmpd3 (FERM and PDZ Domain Containing 3), Si-Frmpd3 (shRNA-Frmpd3), Si-ctrl (shRNA-Control).

To further explore whether Frmpd3 is involved in the regulation of epilepsy susceptibility, we constructed an AAV9 pAAV-hSyn-EGFP-3xFLAG-WPRESiFrmpd3 vector to knock down Frmpd3 in the hippocampal CA1 neurons of mice. The expression efficiency of Frmpd3 knockdown was confirmed after 2 weeks via immunofluorescence (Figure 3A) and Western Blotting (Figure 3B). We then constructed PTZ-induced epilepsy model mice to observe the behavior of the mice and changes in brain electrical activity (Figure 3C). Compared with the Si-ctrl group, the Si-Frmpd3 group presented significantly less seizure progression (Figure 3D), a significantly longer kindling latency (Figure 3E), a lower total number of Grade IV or V seizures (Figure 3F), and a significantly lower incidence of fully kindled seizures (Figure 3G). The LFP results revealed that the amplitude of epileptiform discharge in the Si-Frmpd3 group tended to be lower than that in the Si-ctrl group during the seizure period, and the duration of epileptiform discharge tended to be shorter (Figure 3H). These results suggest that low expression of Frmpd3 in the mouse hippocampus alleviates epilepsy susceptibility in a PTZ-induced epilepsy animal model.

### 3.4. Frmpd3 Combined with GluA2 and GRIP In Vivo

The PDZ domain identifies the postsynaptic membrane of neurotransmitter receptors and transfers cytoplasmic receptors to cell membrane receptors [10]. Therefore, we speculated that Frmpd3, which has a PDZ domain, may participate in the transport of excitatory receptors, which control epileptic seizures.

**Figure 4:**
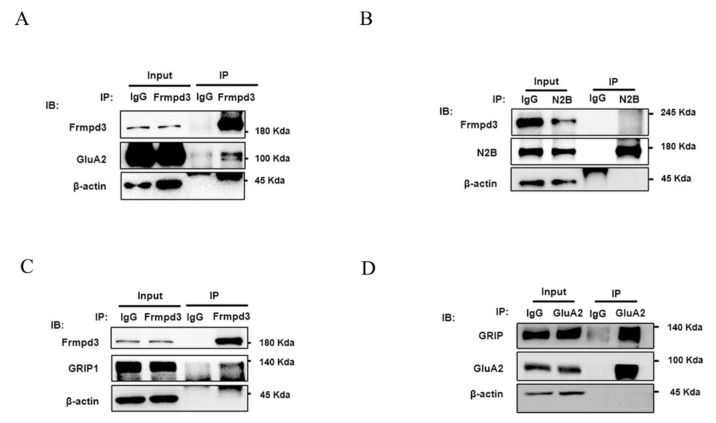
Frmpd3 combined with GluA2and GRIP in vivo. In the hippocampus of the PTZ-induced chronic epilepsy mouse model, Frmpd3 interacted with GluA2 (**A**), Frmpd3 and N2B did not bind to each other (**B**), and Frmpd3 and GRIP (**C**), GRIP and GluA2 combined (**D**) by immunoprecipitation. GluA2 (anti-GluA2 antibody), GRIP (anti-GRIP antibody), N2B (anti-NMDAR2B antibody), Frmpd3 (anti-Frmpd3 antibody), IgG (a negative control antibody).

The excitatory receptor AMPAR subunit GluA2 and the NMDAR subunit N2B are important regulatory targets in epileptic seizures [17,18]. We used immunoprecipitation to explore whether the AMPAR subunit GluA2 and the NMDAR subunit N2B interacts with Frmpd3 in vivo. The results suggested that Frmpd3 interacts with the AMPAR subunit GluA2 (Figure 4A), but not with the NMDAR subunit N2B (Figure 4B) in the hippocampi of PTZ-induced mice.

The glutamate receptor-interacting protein GRIP is currently known to regulate and participate in GluA2 transport as a scaffold protein. By immunoprecipitation in the hippocampus of PTZ-induced mice, we found that Frmpd3 bound to GRIP and that GRIP bound to GluA2 (Figure 4C,D), further indicating that Frmpd3 may be involved in GluA2 transport by binding to GluA2 and GRIP.

### 3.5. Knockdown of the Frmpd3 Protein Decreased the Membrane Expression of the AMPAR Subunit GluA2

**Figure 5:**
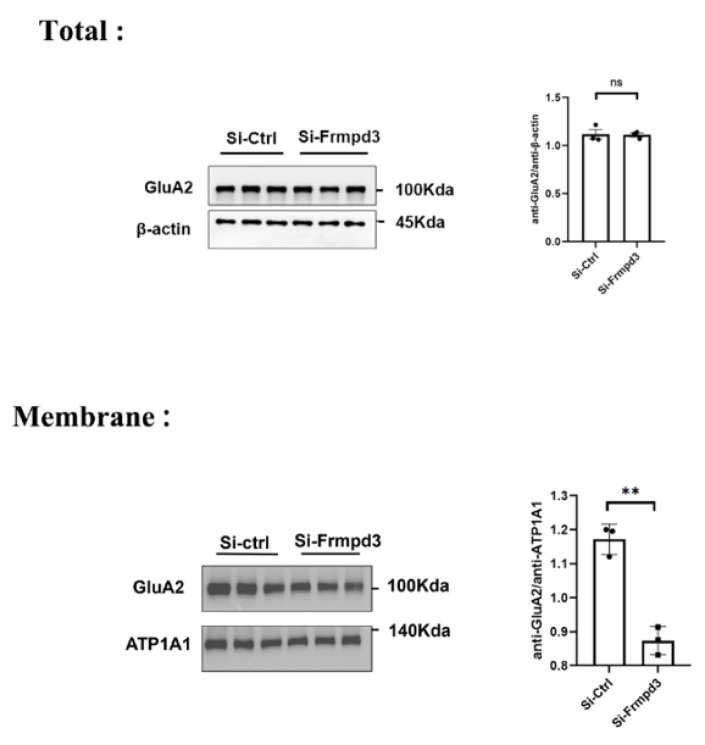
Knockdown of the Frmpd3 protein decreased the membrane expression of the AMPAR subunit GluA2. Two weeks after AAV injection, a PTZ-induced chronic epilepsy model was established, and membrane proteins were extracted from the hippocampal tissue of the mice. In the Si-Frmpd3 group, the total protein level of GluA2 did not change (*n* = 3, unpaired two-tailed Student’s *t*-test; ns = not significant), but the membrane expression level of GluA2 decreased (*n* = 3, unpaired two-tailed Student’s *t*-test; ** *p* < 0.01). GluA2 (anti-GluA2 antibody), ATP1A1 (anti-ATP1A1 antibody), Si-Frmpd3 (shRNA-Frmpd3), Si-ctrl (shRNA-Control).

We extracted total protein and membrane protein from the hippocampus of the Si-Frmpd3 group and the Si-ctrl group for Western blotting. The results indicated that the total GluA2 protein level did not obviously change and that the GluA2 membrane protein level significantly decreased in the Si-Frmpd3 group compared with the Si-ctrl group (Figure 5). Combined with the above experimental results, we hypothesized that the decreased expression level of Frmpd3 may reduce the membrane expression level of GluA2. Frmpd3 may not affect the GluA2 protein expression or degradation.

## 4. Discussion

In our study, we identified a novel scaffold protein, Frmpd3, and provided insights into the mechanism by which Frmpd3 is involved in epilepsy susceptibility and the regulation of pathological neuronal excitability.

Our results demonstrated that the Frmpd3 protein is expressed in hippocampus, cortex, hypothalamus, and cerebellum neurons of mice. The hippocampus, cortex [19], hypothalamus [20], and cerebellum [21] play pivotal roles in the occurrence and development of epilepsy. Notably, the hippocampus, which is particularly vulnerable to damage in temporal lobe epilepsy, serves as a critical region for epileptogenesis [22]. These findings collectively suggest that the Frmpd3 protein is anatomically associated with epilepsy-related brain regions. Furthermore, we observed a significant upregulation of Frmpd3 protein expression in the hippocampus of PTZ-induced epileptic mice. Considering that genetic and protein alterations have been implicated in the development of epileptic phenotypes, we further investigated the potential involvement of Frmpd3 in epileptogenic processes. Through hippocampal-specific knockdown of Frmpd3 expression, we demonstrated a marked suppression of seizure activity, characterized by prolonged seizure latency and reduced seizure severity. These experimental results strongly indicate that Frmpd3 may play a regulatory role in modulating epileptic susceptibility.

Previous studies have identified Frmpd3 localization in excitatory synapses in vitro [13]. Our results also showed that Frmpd3 is distributed within the postsynaptic density (PSD) of excitatory synapses in the hippocampal CA1 region of mice. It is suggested that Frmpd3 may contribute to epileptogenesis through the modulation of excitatory synaptic transmission. The process of synaptic transmission initiates with the release of readily releasable neurotransmitters from presynaptic terminals, followed by their diffusion across the synaptic cleft and subsequent binding to postsynaptic receptors. Importantly, disruptions in any aspect of neurotransmitter receptor biology—including expression, clustering, trafficking, localization, recycling, and degradation—have been established as fundamental pathological mechanisms underlying various neurological disorders, such as dementia, schizophrenia, and epilepsy.

PDZ scaffold proteins, which bind to neurotransmitter receptors and participate in receptor trafficking and anchoring, have been recognized as crucial regulators of synaptic plasticity, learning, memory, cognition, and seizure activity [10]. Through their PDZ domains, these scaffold proteins exhibit the capacity to interact with various neurotransmitter receptors and their distinct subunits. The homologous scaffold protein Frmpd2 has been shown to specifically interact with the C-terminus of the NR2A [15]. Similarly, the scaffold protein GRIP, containing seven PDZ domains, demonstrates specific binding properties: its fourth and fifth PDZ domains directly interact with the C-termini of AMPAR subunits GluA2 and GluA3, respectively [8]. In our current study, we have identified that the scaffold protein Frmpd3 interacts with both the AMPAR subunit GluA2 and GRIP through in vivo immunoprecipitation experiments. These findings suggest that the PDZ domain of Frmpd3 may serve as a critical structural determinant mediating the association between Frmpd3, GluA2, and GRIP.

The presence of the AMPAR subunit GluA2 on the cell membrane plays a crucial regulatory role in controlling calcium permeation and voltage rectification properties of AMPARs. Notably, the absence of the GluA2 subunit leads to excessive calcium influx, which poses a significant risk for both synaptic plasticity impairment and the development of various neurological disorders, particularly epilepsy [23]. Our experimental results demonstrated that Frmpd3 enhances the membrane localization of GluA2 without affecting its total protein expression level in neurons. It is suggested that Frmpd3, through its interactions with both GluA2 and GRIP proteins, facilitates the trafficking of GluA2 from the cytoplasm to the cell membrane, thereby increasing its membrane expression level. These findings highlight the cooperative nature of PDZ scaffold proteins in regulating neurotransmitter receptor trafficking. GRIP-AMPAR interactions are also reported to play a pivotal role in modulating synaptic AMPAR levels at the cell surface by controlling the dynamic trafficking of AMPARs between the cytoplasmic compartment and plasma membrane [8]. The synapse-associated protein SAP97 in mice has been shown to specifically interact with the C-terminus of the AMPAR subunit GluA1 through its PDZ domain [24]. Notably, overexpression of SAP97 can effectively compensate for impaired AMPAR transmission resulting from PSD-95 deficiency. Both proteins belong to the PDZ domain-containing protein family, with PSD-95 serving as another crucial scaffold protein that interacts with AMPARs and forms complexes with SAP97 [8].

**Figure 6:**
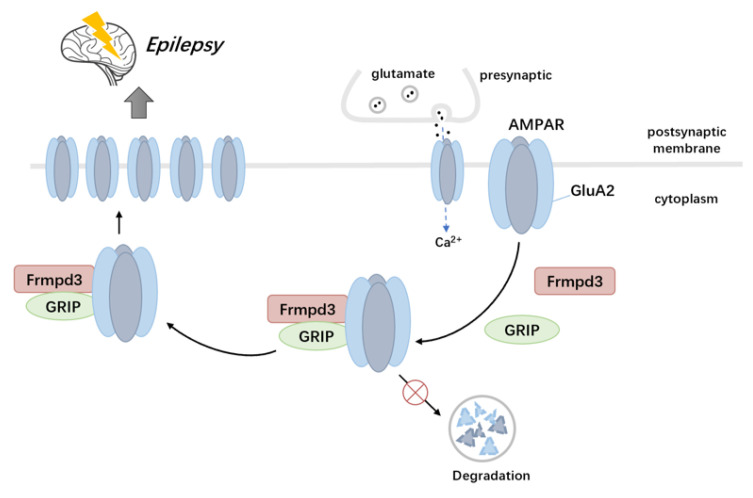
Scaffold protein Frmpd3 modulates epileptic susceptibility. The underlying mechanism appears to involve Frmpd3 mediates GluA2 trafficking from the cytoplasm to the membrane through molecular interactions with GRIP and GluA2, further increases membrane expression of GluA2-containing AMPA receptors, ultimately up-regulates neuronal excitability. Frmpd3 (FERM and PDZ Domain Containing 3), AMPAR (a glutamate receptor), GluA2 (a subunit of AMPAR), GRIP (a scaffolding protein involved in AMPAR transport).

Our study has identified the novel scaffold protein Frmpd3 as a crucial regulator of both seizure activity and AMPAR trafficking through its interactions with GRIP and GluA2. The dysregulation of this scaffold protein-mediated regulatory network leads to impaired excitatory synaptic transmission (Figure 6), which represents a fundamental pathological mechanism underlying epilepsy and related neurological disorders.

## Data Availability

The data will be made available upon request.

## References

[1] Manford M. (2017). Recent advances in epilepsy. J. Neurol..

[2] French J.A. (2007). Refractory epilepsy: Clinical overview. Epilepsia.

[3] Bassani S., Folci A., Zapata J., Passafaro M. (2013). AMPAR trafficking in synapse maturation and plasticity. Cell. Mol. Life Sci..

[4] Diering G.H., Huganir R.L. (2018). The AMPA Receptor Code of Synaptic Plasticity. Neuron.

[5] French J.A., Krauss G.L., Biton V., Squillacote D., Yang H., Laurenza A., Kumar D., Rogawski M.A. (2012). Adjunctive perampanel for refractory partial-onset seizures: Randomized phase III study 304. Neurology.

[6] Nissenkorn A., Kluger G., Schubert-Bast S., Bayat A., Bobylova M., Bonanni P., Ceulemans B., Coppola A., Di Bonaventura C., Feucht M. (2023). Perampanel as precision therapy in rare genetic epilepsies. Epilepsia.

[7] Nakagawa T., Wang X.T., Miguez-Cabello F.J., Bowie D. (2024). The open gate of the AMPA receptor forms a Ca(^2+^) binding site critical in regulating ion transport. Nat. Struct. Mol. Biol..

[8] Cao Y.Y., Wu L.L., Li X.N., Yuan Y.L., Zhao W.W., Qi J.X., Zhao X.Y., Ward N., Wang J. (2023). Molecular Mechanisms of AMPA Receptor Trafficking in the Nervous System. Int. J. Mol. Sci..

[9] Bard L., Sainlos M., Bouchet D., Cousins S., Mikasova L., Breillat C., Stephenson F.A., Imperiali B., Choquet D., Groc L. (2010). Dynamic and specific interaction between synaptic NR2-NMDA receptor and PDZ proteins. Proc. Natl. Acad. Sci. USA.

[10] Kim E., Sheng M. (2004). PDZ domain proteins of synapses. Nat. Rev. Neurosci..

[11] Tan H.L., Queenan B.N., Huganir R.L. (2015). GRIP1 is required for homeostatic regulation of AMPAR trafficking. Proc. Natl. Acad. Sci. USA.

[12] Tan H.L., Chiu S.L., Zhu Q., Huganir R.L. (2020). GRIP1 regulates synaptic plasticity and learning and memory. Proc. Natl. Acad. Sci. USA.

[13] Hu J.H., Yang L., Kammermeier P.J., Moore C.G., Brakeman P.R., Tu J., Yu S., Petralia R.S., Li Z., Zhang P.W. (2012). Preso1 dynamically regulates group I metabotropic glutamate receptors. Nat. Neurosci..

[14] Spiegel I., Mardinly A.R., Gabel H.W., Bazinet J.E., Couch C.H., Tzeng C.P., Harmin D.A., Greenberg M.E. (2014). Npas4 regulates excitatory-inhibitory balance within neural circuits through cell-type-specific gene programs. Cell.

[15] Lu X., Zhang Q., Wang T. (2019). The second PDZ domain of scaffold protein Frmpd2 binds to GluN2A of NMDA receptors. Biochem. Biophys. Res. Commun..

[16] Shimada T., Yamagata K. (2018). Pentylenetetrazole-Induced Kindling Mouse Model. J. Vis. Exp..

[17] Kamenetz F., Tomita T., Hsieh H., Seabrook G., Borchelt D., Iwatsubo T., Sisodia S., Malinow R. (2003). APP processing and synaptic function. Neuron.

[18] Shankar G.M., Li S., Mehta T.H., Garcia-Munoz A., Shepardson N.E., Smith I., Brett F.M., Farrell M.A., Rowan M.J., Lemere C.A. (2008). Amyloid-beta protein dimers isolated directly from Alzheimer’s brains impair synaptic plasticity and memory. Nat. Med..

[19] Galovic M., van Dooren V.Q.H., Postma T.S., Vos S.B., Caciagli L., Borzì G., Cueva Rosillo J., Vuong K.A., de Tisi J., Nachev P. (2019). Progressive Cortical Thinning in Patients With Focal Epilepsy. JAMA Neurol..

[20] Li H.T., Viskaitis P., Bracey E., Peleg-Raibstein D., Burdakov D. (2024). Transient targeting of hypothalamic orexin neurons alleviates seizures in a mouse model of epilepsy. Nat. Commun..

[21] Streng M.L., Krook-Magnuson E. (2021). The cerebellum and epilepsy. Epilepsy Behav..

[22] Ji C., Zhu L., Chen C., Wang S., Zheng L., Li H. (2018). Volumetric Changes in Hippocampal Subregions and Memory Performance in Mesial Temporal Lobe Epilepsy with Hippocampal Sclerosis. Neurosci. Bull..

[23] Wright A., Vissel B. (2012). The essential role of AMPA receptor GluR2 subunit RNA editing in the normal and diseased brain. Front. Mol. Neurosci..

[24] Nikandrova Y.A., Jiao Y., Baucum A.J., Tavalin S.J., Colbran R.J. (2010). Ca^2+^/calmodulin-dependent protein kinase II binds to and phosphorylates a specific SAP97 splice variant to disrupt association with AKAP79/150 and modulate alpha-amino-3-hydroxy-5-methyl-4-isoxazolepropionic acid-type glutamate receptor (AMPAR) activity. J. Biol. Chem..

